# Long-Term Outcomes of Sex Differences in Three-Vessel Coronary Disease with Different Treatment Strategies: A Large Cohort Study

**DOI:** 10.5334/gh.1333

**Published:** 2024-07-03

**Authors:** Jiawen Li, Lin Jiang, Lianjun Xu, Jian Tian, Xinxing Feng, Dong Wang, Yin Zhang, Rutai Hui, Runlin Gao, Lei Song, Jinqing Yuan, Xueyan Zhao

**Affiliations:** 1 Department of Cardiology, National Clinical Research Center for Cardiovascular Diseases, State Key Laboratory of Cardiovascular Disease, Fu Wai Hospital, National Center for Cardiovascular Diseases, Chinese Academy of Medical Sciences and Peking Union Medical College, Beijing 100037, China

**Keywords:** sex differences, women, three-vessel coronary disease, medical therapy, percutaneous coronary intervention, coronary artery bypass grafting surgery

## Abstract

**Aim:**

The information assessing sex differences in outcomes of patients with three-vessel coronary disease (TVD) after different treatment strategies is sparse. This study aimed to investigate long-term outcomes of TVD among women compared with men after medical therapy (MT) alone, percutaneous coronary intervention (PCI), or coronary artery bypass grafting surgery (CABG).

**Methods:**

Consecutive 8943 patients with TVD were enrolled. Associations between sex and all-cause death and major adverse cardiac and cerebrovascular events (MACCE) (all-cause death, myocardial infarction, or stroke) were assessed.

**Results:**

Of the 8943 patients, 1821 (20.4%) were women. During a median follow-up of 6.6 years, women had comparable incidences of all-cause death (16.6% vs. 14.9%, *P* = 0.079) and MACCE (27.2% vs. 26.1%, *P* = 0.320) to men. After multivariable analysis, women showed lower adjusted risks of all-cause death (HR: 0.777; *P* = 0.001) and MACCE (HR: 0.870; *P* = 0.016) than men in the entire cohort. Subgroup analysis revealed that the less all-cause death risk of women relative to men was significant in PCI (HR: 0.702; *P* = 0.009), and CABG groups (HR: 0.708; *P* = 0.047), but not in MT alone group. Lower MACCE risk for women vs. men was significant only in PCI group (HR: 0.821; *P* = 0.037). However, no significant interaction between sex and three strategies was observed for all-cause death (*P* for interaction = 0.312) or MACCE (*P* for interaction = 0.228).

**Conclusions:**

The cardiovascular prognosis of TVD female patients is better than that of men, which has no interaction with the treatment strategies received (MT alone, PCI, or CABG).

## Introduction

Coronary heart disease (CHD) is a gender-specific condition that affects women differently than men. However, women have lower awareness rates and social attention regarding CHD, which poses significant challenges to its prevention and control among female populations. The relationship between sex and prognosis of CHD is still inconclusive. Some earlier studies have revealed that women with CHD have a worse prognosis than men [1,2,3,4], but many studies have indicated that after adjusting for other factors and comorbidities, female sex is not observed to be an independent risk factor for poor prognosis of CHD [5,6,7]. In recent years, some studies have even suggested that women with CHD had a better prognosis than men [8,9,10,11]. The latest research has demonstrated that women with acute chest pain have better short- and long-term death and readmission rates than men [12]. However, most of the current research on the role of sex as a potential cardiovascular risk factor has focused on acute coronary syndrome (ACS) patients [1,2,3,4,5,6,7, 13]. At the same time, the inconsistent findings from different studies have raised the question of whether CHD management should be tailored to sex.

Patients with three-vessel coronary disease (TVD), a subtype of CHD frequently complicated with diffuse and severe lesions, have almost double the mortality rate higher than those with single-vessel disease [14]. However, few studies have examined the sex difference in clinical outcomes among this very high-risk population. Individuals with TVD usually receive one of the following treatments: percutaneous coronary intervention (PCI) revascularization, coronary artery bypass grafting surgery (CABG) revascularization, or medical therapy (MT) alone. It is not clear whether sex influences the prognosis of TVD patients who undergo different treatment strategies. Hence, the goal of the present study was to determine the effect of sex (women relative to men) on long-term outcomes in TVD patients. We investigated the association between sex and median 6.6-year outcomes in the entire cohort and subgroups stratified by three treatment strategies: MT alone, PCI, or CABG, using a large real-world cohort with long follow-up.

## Methods

### Study design and patients

This was a single-center, prospective, observational, real-world, and all-comer study. From April 2004 to February 2011, this study included 8943 patients with TVD without pre-specified exclusion criteria from Fu Wai Hospital (Beijing, China). TVD was defined as stenosis ≥50% confirmed by angiography in all three main epicardial coronary arteries (left anterior descending, left circumflex, and right coronary arteries) with or without left main coronary involvement. The initial purpose of this large-scale prospective cohort was to assess the long-term efficacy of three distinct treatments (MT alone, PCI, or CABG) on TVD.

The study complied with the principles of the Declaration of Helsinki and was approved by the Review Board of Fu Wai Hospital. Written informed consent was obtained from all participants.

### Treatment strategies

In the present study, each patient received one of three treatment strategies (MT alone, PCI, or CABG) considering current practice guidelines, the judgments of cardiology team, and the preferences of patients.

Regarding the patients undergoing PCI, they were all given aspirin in addition to clopidogrel. Those with selective PCI without taking long-term aspirin and clopidogrel were given 300 mg aspirin and 300 mg clopidogrel orally at least 24 h before the operation. After finishing the operation, they were administered aspirin 100 mg once daily indefinitely, and clopidogrel 75 mg once daily for at least one year.

Regarding the patients undergoing CABG, left internal mammary artery was regularly used as a graft to left anterior descending artery, followed by venous grafts to other coronary branches with conventional bypass methods. Surgeons conducted the operation, and whether it was done on- or off-pump was up to the operator.

Regarding the patients who received MT alone, the individual medication therapy consisted of a titrated regimen with nitrates, aspirin, clopidogrel, statins, β-blockers, calcium channel blockers, angiotensin-converting enzyme inhibitors, or a combination of these drugs, unless they had contraindications.

### Clinical outcomes

The primary endpoint was all-cause death. All deaths were considered cardiac unless an unequivocal non-cardiac cause could be established. The secondary endpoint was major adverse cardiac and cerebrovascular events (MACCE), a composite of all-cause death, myocardial infarction (MI), and stroke. All endpoint events were adjudicated centrally by an independent group of cardiologists. Investigator training, blinded questionnaire filling, and telephone recording were performed to obtain high-quality data. The diagnostic criteria for MI were in accordance with the third global standard definition. Stroke included ischemic stroke and hemorrhagic stroke. Follow-ups were conducted through clinic visits, follow-up letters, or telephone interviews. Each patient had at least one follow-up visit and the last follow-up was in 2016. Patients who were lost to follow-up were censored at the last available contact.

### Statistical analysis

Continuous variables were reported as means ± standard deviation or medians (interquartile range) and were compared by Student’s *t* test or nonparametric test. Categorical variables were expressed as frequency (%) and compared by χ^2^ or Fisher exact test. Missing values were imputed using the median for continuous variables or the mode for categorical variables, except for the SYNTAX score. Detailed information on the numbers of missing values and descriptive statistics are shown in Table S1. Univariable and multivariable Cox proportional hazard regressions were performed to calculate the hazard ratio (HR) and 95% confidence interval (CI) and evaluate the associations between sex (women relative to men) and long-term clinical outcomes. The covariates getting involved in the multivariable model in an all-enter way were: sex, age, body mass index, diabetes mellitus, hyperlipidemia, chronic obstructive pulmonary disease, peripheral artery disease, previous MI, previous stroke, previous PCI, previous CABG, ACS, left main coronary involvement, left ventricular ejection fraction, creatinine clearance, baseline SYNTAX score, and treatment strategy (including MT alone, PCI, or CABG). The variables were included in the multivariable model based on the clinical relevance and their associations with the primary outcome. The crude and multivariable-adjusted data from the Cox regression analyses were also plotted as 10-year cumulative events curves for men and women in the entire cohort. The crude survival curves were drawn by Kalpan–Meier method. The adjusted survival curves were drawn by separating lines for sex (women relative to men) in Cox Regression: Plots of SPSS 23.0. All statistical analyses were performed at a significance level of two-sided 0.05. Statistical analyses were performed via SPSS 23.0 (IBM Corporation, Armonk, New York), and R Programming Language version 4.0.3 (R Core Team, 2014).

## Results

### Baseline characteristics

**Figure 1:**
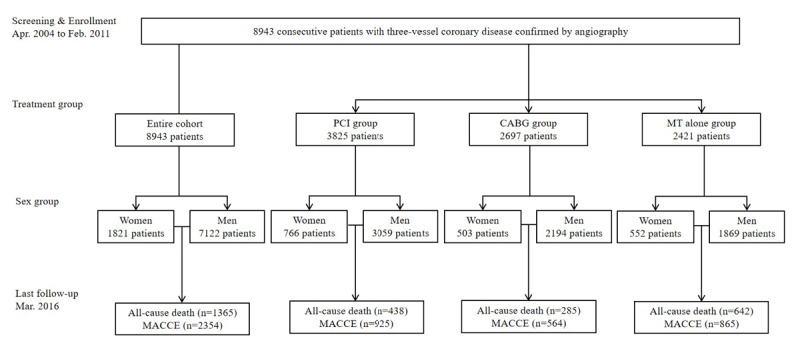
**The flow chart of the study**. MT, medical therapy; PCI, percutaneous coronary intervention; CABG, coronary artery bypass grafting; MACCE: major adverse cardiac and cerebrovascular events.

The resulting 8943 (61.1 ± 10.0 years) eligible patients were finally included (Figure 1). Of these, 1821 (20.4%) individuals were women, 5437 (60.8%) presented as ACS, and 3506 (39.2%) presented as stable angina pectoris. Compared with men, women were older, had a lower body mass index, had higher rates of hypertension and diabetes mellitus, had lower rates of previous MI, previous PCI, and previous CABG, had fewer current/former smokers, had fewer left main involvement, had higher levels of left ventricular ejection fraction, total cholesterol, high-density lipoprotein cholesterol, and low-density lipoprotein cholesterol, had a lower level of creatinine clearance, were more likely to present as ACS, and were more likely to receive angiotensin receptor blocker and calcium channel blocker treatment (Table 1). Most of the comorbidities (hyperlipidemia, chronic obstructive pulmonary disease, and peripheral artery disease), evidence-based medication at discharge (aspirin, clopidogrel, angiotensin II receptor blockers, β-blockers, and statins), and severity of lesion (SYNTAX score) were comparable between women and men (Table 1). The baseline characteristics of women relative to men in the three treatment groups are provided in Table S2.

**Table 1:** Baseline Characteristics.


PARAMETERS	ENTIRE COHORT (n = 8943)	MEN (n = 7122)	WOMEN (n = 1821)	P VALUE

Demographic characteristics				

Age (years)	61.1 ± 10.0	60.0 ± 10.1	65.3 ± 8.1	<0.001

BMI (kg/m^2^)	25.8 ± 3.1	25.9 ± 3.0	25.5 ± 3.3	<0.001

Past medical history				

Previous MI	3184 (35.6)	2735 (38.4)	449 (24.7)	<0.001

Previous stroke	888 (9.9)	699 (9.8)	189 (10.4)	0.500

Hypertension	6055 (67.7)	4628 (65.0)	1427 (78.4)	<0.001

Hyperlipidaemia	5005 (56.0)	3958 (56.0)	1047 (57.5)	0.148

Diabetes	3117 (34.9)	2333 (32.8)	784 (43.1)	<0.001

COPD	100 (1.1)	81 (1.1)	19 (1.0)	0.830

PAD	690 (7.7)	546 (7.7)	144 (7.9)	0.768

Previous PCI	1131 (12.7)	971 (13.6)	160 (8.8)	<0.001

Previous CABG	307 (3.4)	259 (3.6)	48 (2.6)	0.043

Current/former smoker	4981 (55.7)	4711 (66.2)	270 (14.8)	<0.001

Clinical presentation				0.002

SAP	3506 (39.2)	2849 (40.0)	657 (36.1)	

ACS	5437 (60.8)	4273 (60.0)	1164 (63.9)	

LVEF (%)	58.2 ± 9.8	58.0 ± 9.9	59.2 ± 9.5	<0.001

Laboratory examination				

TC (mmol/L)	4.6 ± 1.1	4.5 ± 1.0	4.9 ± 1.2	<0.001

HDL-C (mmol/L)	1.0 ± 0.3	1.0 ± 0.3	1.1 ± 0.3	<0.001

LDL-C (mmol/L)	2.6 ± 0.9	2.5 ± 0.9	2.7 ± 0.9	<0.001

CCr (mL/min)^a^	85.5 ± 26.8	88.7 ± 26.7	72.7 ± 23.1	<0.001

Angiographic characteristics				

Left main involvement	2063 (23.1)	1684 (23.7)	379 (20.8)	0.011

SYNTAX score^b^	25.3 ± 11.2	25.3 ± 11.5	25.1 ± 10.0	0.504

Treatment strategies				

MT alone	2421 (27.1)	1869 (26.2)	552 (30.3)	0.001

PCI	3825 (42.8)	3059 (43.0)	766 (42.1)	

CABG	2697 (30.2)	2194 (30.8)	503 (27.6)	

Medication at discharge				

Aspirin	8540 (95.5)	6803 (95.5)	1737 (95.4)	0.855

Clopidogrel	4670 (52.2)	3739 (52.5)	931 (51.1)	0.307

ACEI	3309 (37.0)	2664 (37.4)	645 (35.4)	0.124

ARB	1358 (15.2)	1001 (14.1)	357 (19.6)	<0.001

Nitrate	8288 (92.7)	6614 (92.9)	1674 (91.9)	0.170

Beta-blocker	7858 (87.9)	6242 (87.6)	1616 (88.7)	0.215

CCB	3229 (36.1)	2440 (34.3)	789 (43.3)	<0.001

Statin	5982 (66.9)	4737 (66.5)	1245 (68.4)	0.140


Values are presented as mean ± standard deviation or number (%). ACEI, angiotensin-converting enzyme inhibitors; ACS, acute coronary syndrome; ARB, angiotensin II receptor blockers; BMI, body mass index; CABG, coronary artery bypass grafting; CCB, calcium channel blocker; CCr, creatinine clearance; COPD, chronic obstructive pulmonary disease; HDL-C, high-density lipoprotein cholesterol; LDL-C, low-density lipoprotein cholesterol; TC, total cholesterol; LVEF, left ventricular ejection fraction; MI, myocardial infarction; MT, medical therapy; PAD, peripheral artery disease; PCI, percutaneous coronary intervention; SAP, stable angina pectoris. ^a^Calculated using the Cockcroft and Gault formula. ^b^Calculated using an online calculator (http://www.syntaxscore.com) by a dedicated research group blinded to the clinical data.

### Sex differences in long-term outcomes of TVD in the entire cohort

**Figure 2:**
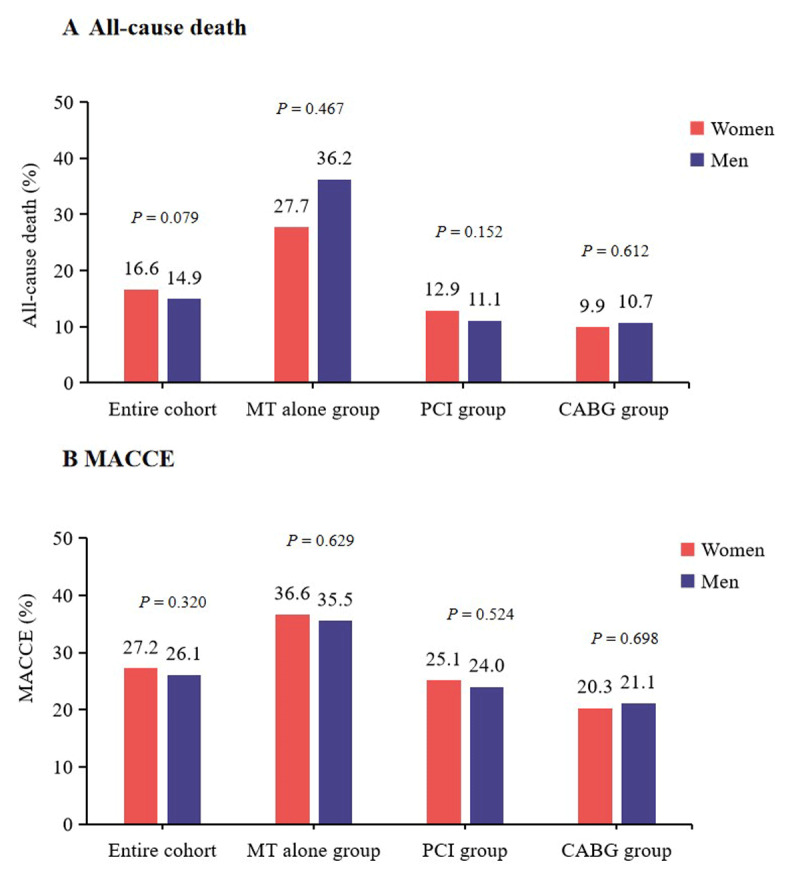
**Incidences of clinical outcomes according to sex in the entire cohort and treatment strategy stratification**. Incidence of all-cause death **(A)** and incidence of MACCE **(B)**. MT, medical therapy; PCI, percutaneous coronary intervention; CABG, coronary artery bypass grafting; MACCE: major adverse cardiac and cerebrovascular events.

Over a median follow-up of 6.6 years (interquartile range 5.1–8.6) with a response rate of 80.5% (Table S3), 1365 (15.3%) all-cause death, 2354 (26.3%) MACCE (Figure 1), 515 (5.8%) MI, and 642 (7.2%) stroke occurred. The incidences of all-cause death (16.6% vs. 14.9%, *P* = 0.079), MACCE (27.2% vs. 26.1%, *P* = 0.320), MI (5.2% vs. 5.9%, *P* = 0.221), and stroke (7.9% vs. 7.9%, *P* = 0.212) were comparable in women and men groups in the entire cohort (Figure 2 and Table S4).

**Figure 3:**
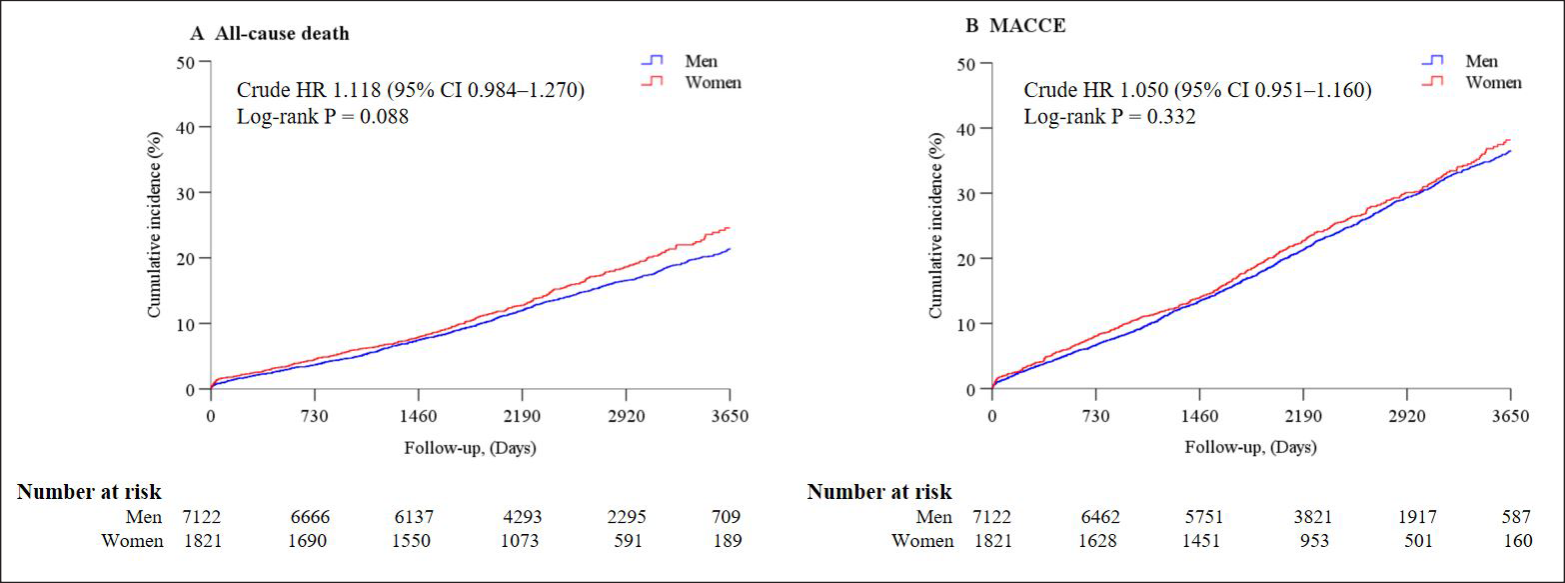
**Ten-year cumulative event curves between men and women in the entire cohort from crude data**. Crude cumulative incidence of all-cause death **(A)**. Crude cumulative incidence of MACCE **(B)**. MACCE: major adverse cardiac and cerebrovascular events.

Kaplan–Meier curve analysis showed that the 10-year cumulative all-cause death rates in men and women were 21.5% and 24.6%, respectively (log-rank *P* = 0.088); the 10-year cumulative MACCE rates in men and women were 36.6% and 38.2%, respectively (log-rank *P* = 0.332) (Figure 3).

**Figure 4:**
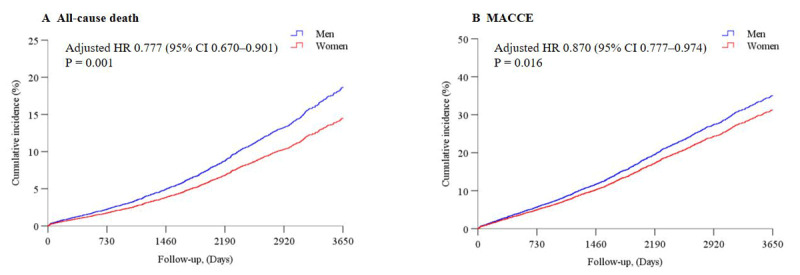
**Ten-year cumulative events curves between men and women in the entire cohort from multivariable-adjusted data**. Adjusted cumulative incidence of all-cause death **(A)**. Adjusted cumulative incidence of MACCE **(B)**. MACCE: major adverse cardiac and cerebrovascular events; HR, hazard ratio; CI, confidence interval. The covariates adjusted were: sex, age, body mass index, diabetes, hyperlipidemia, chronic obstructive pulmonary disease, peripheral artery disease, previous myocardial infarction, previous stroke, previous PCI, previous CABG, acute coronary syndrome, left main coronary disease, left ventricular ejection fraction, creatinine clearance, baseline SYNTAX score, and treatment strategies (MT alone, PCI, or CABG).

Multivariate Cox regression showed that women had better prognoses than men in the entire cohort with a 22.3% lower risk of all-cause death (HR 0.777, 95% CI 0.670–0.901) and a 13.0% lower risk of MACCE (HR 0.870, 95% CI 0.777–0.974) (Table 2, Figure 4, and Table S5). The risk of MI and stroke was similar in women compared with men in the entire cohort (Table S6).

**Table 2:** The Effect of Sex on Long-term Clinical Outcomes in Patients with TVD in the Entire Cohort and Treatment Strategy Stratification.


CLINICAL OUTCOMES	WOMEN VS. MEN	ADJUSTED HR (95% CI)	P VALUE

All-cause death	Entire Cohort	0.777 (0.670–0.901)	0.001

	MT alone group	0.872 (0.706–1.077)	0.204

	PCI group	0.702 (0.538–0.915)	0.009

	CABG group	0.708 (0.504–0.996)	0.047

	P for interaction		0.312

MACCE	Entire Cohort	0.870 (0.777–0.974)	0.016

	MT alone group	0.916 (0.764–1.099)	0.345

	PCI group	0.821 (0.681–0.989)	0.037

	CABG group	0.922 (0.730–1.166)	0.499

	P for interaction		0.228


MT, medical therapy; PCI, percutaneous coronary intervention; CABG, coronary artery bypass grafting; MACCE: major adverse cardiac and cerebrovascular events; HR, hazard ratio; CI, confidence interval.

### Sex differences in long-term outcomes of TVD stratified by treatment strategies

Of the entire cohort, there were 2421 (27.1%) patients treated with MT alone, 3825 (42.8%) patients treated with PCI, and 2697 (30.2%) patients treated with CABG (Table 1). For all-cause death, the incidence of women compared with men was similar in the MT alone group (27.7% vs. 36.2%, *P* = 0.467), in the PCI group (12.9% vs. 11.1%, *P* = 0.152), and in the CABG group (9.9% vs. 10.7%, *P* = 0.612) (Figure 2). For MACCE, the incidence of women compared with men was also similar after MT alone (36.6% vs. 35.5%, *P* = 0.629), PCI (25.1% vs. 24.0%, *P* = 0.524), and CABG (20.3% vs. 21.1%, *P* = 0.698) (Figure 2). For MI and stroke, the incidence of women relative to men was comparable in the MT alone, PCI and CABG groups (Table S4).

Subgroup analysis showed that among patients treated with MT alone, women had comparable adjusted risks of long-term outcomes (all-cause death and MACCE) to men. In regard to patients who underwent PCI, women had better prognoses for all-cause death (HR 0.702, 95% CI 0.538–0.915) and MACCE (HR 0.821, 95% CI 0.681–0.989) than men. As for patients with CABG, the prognosis for all-cause death (HR 0.708, 95% CI 0.504–0.996) was better in women than men, and women’s risk of MACCE was similar to men’s (Table 2). However, no significant interaction between sex and these three strategies was observed for all-cause death (*P* for interaction = 0.312) or MACCE (*P* for interaction = 0.228) (Table 2). The risk of MI and stroke was similar in women compared with men in the MT alone, PCI and CABG groups (Table S6).

## Discussion

In this large-scale, real-world study of 8943 TVD patients with a median 6.6-year follow-up, we assessed the impact of sex (women vs. men) on long-term prognosis in the entire cohort, and whether different treatment strategies (MT alone, PCI or CABG) has significant interaction with sex. The major findings were as follows: (1) in the entire cohort, the adjusted-risks of all-cause death and MACCE were lower in female subjects than the male; (2) no significant sex-treatment strategy interaction was observed for all-cause death or MACCE, although the subgroup analysis revealed that in the PCI group, women had lower adjusted-risks of all-cause death and MACCE than men; in the CABG group, female patients experienced a lower adjusted-risk of all-cause death than and an equal adjusted-risk of MACCE to their male counterparts; in the MT alone group, female patients experienced equal adjusted-risks of all-cause death and MACCE to the male.

### Sex differences in overall TVD patients

TVD is a high-risk subtype of CHD and often associated with poor outcomes. Our research demonstrated that women with TVD had more favorable long-term prognoses than their male counterparts. It is well established that women and men differ in hormone levels, body weight, coronary microvascular disease, and other factors, but the mechanisms underlying sex differences in clinical outcomes are largely unknown. Notably, women in the present study were older and had more comorbidities than men, which may lead to an increased risk of adverse outcomes, but interestingly, after adjusting for confounding factors, women exhibited a 22.3% lower risk of all-cause death than men. In addition, previous studies on sex differences in TVD patients focused on those who underwent revascularization only. However, in the real world, they often receive one of the following treatments: MT alone, PCI, or CABG. This large-scale, real-world study encompassed patients receiving revascularization and medications alone. Our study appears to be the first to report superior long-term outcomes in women over men in the entire cohort of TVD, providing evidence for sex differences with valuable clinical implications.

### Sex differences in TVD patients after PCI or CABG

A significant proportion of patients with TVD require PCI or CABG to improve chest pain symptoms or clinical outcomes. Sex has been established as an important factor to be considered in the decision-making process of revascularization [15]. The present study revealed that the adjusted risk of long-term adverse cardiac outcomes was significantly lower in female patients with TVD than the male after revascularization (both PCI and CABG). Studies assessing the long-term outcomes of TVD patients after revascularization from a sex perspective are scarce and inconsistent. Yamaji et al. [8] reported in a large registry study of patients who underwent PCI (46% multi-vessel) that women had a lower 10-year risk of adverse cardiac outcomes compared to men even after multivariate adjustment, despite women had a higher cardiovascular risk than men at baseline. They also found in TVD patients after revascularization that women had better clinical outcomes in long-term (5–10 years) all-cause death than men, but they aimed to explore whether the effect of revascularization (PCI vs. CABG) on the prognosis is affected by gender, rather than the effect of gender (women vs. men) on the prognosis in TVD patients with revascularization [9]. In addition, a subanalysis of the BARI study [10] showed a significant reduction in the 5.4-year risk of death in women in patients with multi-vessel coronary disease (MVD), but this study encompassed a limited number of TVD patients and was published in the last century, so its validity may be outdated. In general, these studies were consistent with our findings [8,9,10].

Besides, there were also studies inconsistent with our findings. The SYNTAX trial [16] (60.8% TVD) showed a higher 4-year adjusted risk of all-cause death in women treated with PCI than in men, and a similar risk between women and men treated with CABG, but in a subsequent 10-year follow-up, female sex was no longer an independent predictor of all-cause mortality [17]. It should be noted that in real-world settings, women tend to choose conservative treatment over invasive ones, implying that sex-related results from these randomized trials might lack representativeness. At the same time, there were also some observational studies that reported no significant difference in long-term outcomes of MVD between men and women in patients who received PCI or CABG [18,19,20]. According to the study mentioned above, sex-induced differences in clinical outcomes are currently primarily attributed to the length of follow-up, composite of endpoint, baseline clinical or anatomical characteristics, intervention or surgery, race, and stable CHD or ACS [1,2,3,4, 13, 21].

### Sex differences in TVD patients after medical therapy alone

In the real world, patients with TVD who choose MT alone may have mild coronary stenosis not requiring revascularization; revascularization contraindicated due to CAD complexity or lack of myocardial viability; or patient unwillingness to undergo revascularization. Although the prognosis of MT alone may not be as favorable as revascularization in patients with MVD [22], it is particularly important to discuss with patients and respect their preferences when there is no clear optimal strategy. Our study reported that the MT alone subgroup had the same effect as the entire cohort. Of notice, women tend to prefer medication, so it is of great significance to explore sex-specific prognosis in this population. To the best of our knowledge, most studies focused on TVD patients after PCI and CABG, leaving sex differences in those treated with MT alone unexplored.

In terms of pharmacokinetics, compared with men, women have lower drug utilization, smaller drug distribution volume, slower drug metabolism, and slower renal excretion rate [23]. Moreover, the enrollment of women has long been underrepresented in the vast majority of cardiovascular drug clinical trials. In clinical practice, physicians rarely consider the effect of sex on cardiovascular drug efficacy, which possibly led to suboptimal treatment for women. Therefore, in future clinical practice, it may be necessary to provide different schemes to men and women in terms of the optimal intensity and dosage of medical treatment in TVD patients.

### Clinical implications

In cardiovascular disease, gender differences are the focus of much attention. Although women have a low awareness of CHD and are more likely to have more comorbidities, the present observational study, with the largest TVD patients cohort (*n* = 8943), showed that the female sex was a protective factor for cardiovascular prognosis among TVD patients after revascularization. Among TVD patients treated with PCI and CABG, the adjusted risk of all-cause death was 29.8% and 29.2% lower in women than men, respectively. This finding has important clinical implications. First, it may help to map more accurate treatments for women, thereby improving their prognosis and quality of life. Second, it could eliminate the gender bias that some clinicians may have during the treatment due to the lack of in-depth understanding of female-specific cardiovascular characteristics, which could result in inappropriate treatment. In addition, understanding the protective effect of female sex on long-term prognosis may help to boost treatment confidence and compliance of female patients and to reduce anxiety. Therefore, we should further strengthen the research and exploration in this field to provide better medical services for female patients with cardiovascular disease.

### Study limitations

Our study also had some limitations. First, this was a single-center, observational study. Therefore, this study has the inherent defects of an observational study. Second, nearly 20% of patients were lost to follow-up, which may limit the reliability and generalizability of our findings. Third, even though we attempted to adjust as many important confounding factors as possible, the study could still suffer from residual confounding such as other procedural characteristics. Last, the proportion of patients using clopidogrel at discharge in the entire cohort in our study is relatively low, likely due to the inclusion of a larger number of patients treated with MT alone and CABG. These patients were mostly treated with single antiplatelet therapy at discharge, based on the medication guidelines, patient preferences, clinical experience, and medication habits at that time.

## Conclusions

This large-sample and real-world study evaluated sex-related differences in long-term (median 6.6-year) clinical outcomes of patients with TVD in China. In this high-risk population, women had a lower adjusted risk of long-term clinical outcomes than their male counterparts after revascularization or medical therapy alone. The study highlighted the need for further research and gender-specific improvement initiatives to reduce sex differences.

## Data Accessibility Statement

Due to ethical restrictions related to the consent given by subjects at the time of study commencement, our datasets are available from the corresponding author upon reasonable request after permission of the Institutional Review Board of State Key Laboratory of Cardiovascular Disease, Fu Wai Hospital, National Center for Cardiovascular Diseases.

## Additional File

The additional file for this article can be found as follows:

10.5334/gh.1333.s1 Supplementary Tables. Tables S1 to S6.

## Figures and Tables

**Table:** 

ACS	acute coronary syndrome
CABG	coronary artery bypass grafting surgery
CHD	coronary heart disease
CI	confidence interval
HR	hazard ratio
MVD	multi-vessel coronary disease
MT	medical therapy
MACCE	major adverse cardiac and cerebrovascular events
MI	myocardial infarction
PCI	percutaneous coronary intervention
TVD	three-vessel coronary disease
