# Supplementary material for: Long-Term Outcomes of Sex Differences in Three-Vessel Coronary Disease with Different Treatment Strategies: A Large Cohort Study
Source: Glob Heart. 2024 Jul 3;19(1):57. doi: 10.5334/gh.1333 (PMC11225599; doi:10.5334/gh.1333)
Supplement: Supplementary Tables. — Tables S1 to S6. [file gh-19-1-1333-s1.pdf]

Table S1. Number of Missing Values and Corresponding Dispositions.

	No. of Missing value	Disposition
Body mass index	85 (1.0%)	Median imputation
Previous stroke	24 (0.3%)	Mode imputation
Hypertension	9 (0.1%)	Mode imputation
Hyperlipidemia	23 (0.3%)	Mode imputation
Current/former smoker	14 (0.2%)	Mode imputation
Left ventricular ejection fraction	133 (1.5%)	Median imputation
Total cholesterol	79 (0.9%)	Median imputation
High-density lipoprotein cholesterol	80 (0.9%)	Median imputation
Low-density lipoprotein cholesterol	79 (0.9%)	Median imputation
Creatinine clearance	131 (1.5%)	Median imputation
SYNTAX score	270 (3.0%)	Undisposed

Table S2. Baseline Characteristics in the Entire Cohort.

**Parameters**	**MT alone (n = 2421)**			**PCI (n = 3825)**			**CABG (n = 2697)**		
	**Men (n = 1869)**	**Women (n = 552)**	**P Value**	**Men (n = 3059)**	**Women (n = 766)**	**P Value**	**Men (n = 2194)**	**Women (n = 503)**	**P Value**
Demographic characteristics									
Age (years)	61.96 ± 10.50	66.80 ± 7.88	<0.001	58.39 ± 10.44	65.15 ± 8.53	<0.001	60.53 ± 8.89	63.69 ± 7.51	<0.001
BMI (kg/m^2)	25.71 ± 2.99	25.37 ± 3.55	0.025	26.10 ± 2.98	25.60 ± 3.24	<0.001	25.81 ± 2.95	25.48 ± 3.05	0.022
Past medical history									
Hypertension	1216 (65.06)	439 (79.53)	<0.001	1975 (64.56)	600 (78.33)	<0.001	1437 (65.50)	388 (77.14)	<0.001
Hyperlipidaemia	1021 (54.63)	335 (60.69)	0.013	1774 (57.99)	423 (55.22)	0.178	1163 (53.01)	289 (57.46)	0.079
Diabetes	660 (35.31)	253 (45.83)	<0.001	977 (31.94)	328 (42.82)	<0.001	696 (31.72)	203 (40.36)	<0.001

COPD	33 (1.77)	6 (1.09)	0.357	28 (0.92)	10 (1.31)	0.441	20 (0.91)	3 (0.60)	0.671
PAD	180 (9.63)	49 (8.88)	0.653	118 (3.86)	28 (3.66)	0.876	248 (11.30)	67 (13.32)	0.233
Current/former smoker	1246 (66.67)	99 (17.93)	<0.001	2063 (67.44)	111 (14.49)	<0.001	1402 (63.90)	60 (11.93)	<0.001
Previous MI	839 (44.89)	172 (31.16)	<0.001	1012 (33.08)	151 (19.71)	<0.001	884 (40.29)	126 (25.05)	<0.001
Previous stroke	223 (11.93)	62 (11.23)	0.709	256 (8.37)	72 (9.40)	0.401	220 (10.03)	55 (10.93)	0.600
Previous PCI	331 (17.71)	61 (11.05)	<0.001	426 (13.93)	63 (8.22)	<0.001	214 (9.75)	36 (7.16)	0.084
Previous CABG	152 (8.13)	25 (4.53)	0.006	94 (3.07)	23 (3.00)	1	13 (0.59)	0 (0.00)	0.170
Clinical presentation									
SAP	706 (37.77)	202 (36.59)	0.650	1100 (35.96)	239 (31.20)	0.015	1043 (47.54)	216 (42.94)	0.070
ACS	1163 (62.23)	350 (63.41)		1959 (64.04)	527 (68.80)		1151 (52.46)	287 (57.06)	
Laboratory examination									

LVEF (%)	54.83 ± 11.76	57.15 ± 11.22	<0.001	59.67 ± 8.55	60.28 ± 8.16	0.094	58.33 ± 9.32	59.71 ± 9.03	0.003
TC (mmol/L)	4.48 ± 1.00	4.89 ± 1.18	<0.001	4.52 ± 1.02	4.86 ± 1.09	<0.001	4.53 ± 1.03	4.97 ± 1.25	<0.001
HDLC (mmol/L)	1.03 ± 0.24	1.13 ± 0.25	<0.001	1.02 ± 0.27	1.15 ± 0.28	<0.001	1.01 ± 0.24	1.11 ± 0.26	<0.001
LDLC (mmol/L)	2.51 ± 1.12	2.70 ± 0.88	<0.001	2.50 ± 0.79	2.64 ± 0.83	<0.001	2.53 ± 0.83	2.78 ± 0.99	<0.001
CCr (mL/min)[a]	84.80 ± 27.78	70.27 ± 24.78	<0.001	92.57 ± 26.82	72.63 ± 22.42	<0.001	86.70 ± 24.67	75.46 ± 21.75	<0.001
Angiographic characteristics									
Left main involvement	505 (27.02)	127 (23.01)	0.067	351 (11.47)	87 (11.36)	0.978	828 (37.74)	165 (32.80)	0.043
SYNTAX score[b]	25.22 ± 12.82	26.01 ± 11.18	0.192	21.44 ± 8.71	21.87 ± 8.61	0.223	30.76 ± 11.45	29.02 ± 9.08	0.001
Medication at discharge									

Aspirin	1731 (92.62)	513 (92.93)	0.873	3003 (98.17)	747 (97.52)	0.310	2069 (94.30)	477 (94.83)	0.721
Clopidogrel	677 (36.22)	189 (34.24)	0.422	2861 (93.53)	704 (91.91)	0.130	201 (9.16)	38 (7.55)	0.291
ACEI	933 (49.92)	241 (43.66)	0.011	1501 (49.07)	349 (45.56)	0.090	230 (10.48)	55 (10.93)	0.829
ARB	351 (18.78)	148 (26.81)	<0.001	594 (19.42)	192 (25.07)	0.001	56 (2.55)	17 (3.38)	0.379
Nitrate	1719 (91.97)	507 (91.85)	0.994	2830 (92.51)	697 (90.99)	0.184	2065 (94.12)	470 (93.44)	0.634
Beta-blocker	1614 (86.36)	500 (90.58)	0.011	2704 (88.39)	681 (88.90)	0.741	1924 (87.69)	435 (86.48)	0.505
CCB	799 (42.75)	292 (52.90)	<0.001	1257 (41.09)	388 (50.65)	<0.001	384 (17.50)	109 (21.67)	0.034
Statin	1621 (86.73)	484 (87.68)	0.610	2783 (90.98)	683 (89.16)	0.142	333 (15.18)	78 (15.51)	0.907

Values are presented as mean ± standard deviation or number (%).

ACEI, angiotensin-converting enzyme inhibitors; ACS, acute coronary syndrome; ARB, angiotensin II receptor blockers; BMI, body mass index; CABG, coronary artery bypass grafting; CCB, calcium channel blocker; CCr, creatinine clearance; COPD, chronic obstructive pulmonary disease; HDL-C, high-density lipoprotein cholesterol; LDL-C, low-density lipoprotein cholesterol; TC, total cholesterol; LVEF, left ventricular

ejection fraction; MI, myocardial infarction; MT, medical therapy; PAD, peripheral artery disease; PCI, percutaneous coronary intervention; SAP, stable angina pectoris.

[a]Calculated using the Cockcroft and Gault formula.

[b]Calculated using an online calculator (http://www.syntaxscore.com) by a dedicated research group blinded to the clinical data.

Table S3. Baseline Characteristics of Participants Who Were Followed Up and Those Who Were Lost To Follow-Up.

Parameter	Followed (n = 7205)	Lost to follow-up (n =1738)	P value
Demographic characteristics			
Age (years)	60.70 ± 9.87	62.54 ± 10.25	<0.001
BMI (kg/m^2)	25.89 ± 3.02	25.58 ± 3.15	<0.001
Male (%)	5734 (79.58)	1388 (79.86)	0.822
Past medical history			
Previous MI	2461 (34.16)	723 (41.60)	<0.001
Previous stroke	675 (9.37)	213 (12.26)	<0.001
Hypertension	4889 (67.86)	1166 (67.09)	0.558
Hyperlipidaemia	4165 (57.81)	840 (48.33)	<0.001
Diabetes	2442 (33.89)	675 (38.84)	<0.001
COPD	78 (1.08)	22 (1.27)	0.600
PAD	544 (7.55)	146 (8.40)	0.253
Previous PCI	908 (12.60)	223 (12.83)	0.828
Previous CABG	246 (3.41)	61 (3.51)	0.902
Current/former smoker	4011 (55.67)	970 (55.81)	0.396
Clinical presentation			

SAP	3506 (39.20)	2860 (39.69)	0.056
ACS	4345 (60.31)	1092 (62.83)	
LVEF (%)	58.69 ± 9.56	56.40 ± 10.67	<0.001
Laboratory examination			
TC (mmol/L)	4.58 ± 1.05	4.64 ± 1.09	0.019
HDL-C (mmol/L)	1.04 ± 0.26	1.04 ± 0.24	0.237
LDL-C (mmol/L)	2.54 ± 0.92	2.59 ± 0.82	0.037
CCr (mL/min)[a]	86.49 ± 26.59	81.16 ± 27.00	<0.001
Angiographic characteristics			
Left main involvement	1647 (22.86)	416 (23.94)	0.355
SYNTAX score[b]	24.98 ± 11.23	26.44 ± 10.94	<0.001
Medication at discharge			
Aspirin	6912 (95.93)	1628 (93.67)	<0.001
Clopidogrel	3829 (53.14)	841 (48.39)	<0.001
ACEI	2622 (36.39)	687 (39.53)	0.016
ARB	1079 (14.98)	279 (16.05)	0.277
Nitrate	6685 (92.78)	1603 (92.23)	0.46
Beta-blocker	6340 (87.99)	1518 (87.34)	0.48
CCB	2549 (35.38)	680 (39.13)	0.004
Statin	4802 (66.65)	1180 (67.89)	0.336

Meanings of the abbreviations are identical to those in Table S2.

Table S4. Incidences of MI and Stroke According to Sex in the Entire Cohort and Treatment Strategy Stratification.

	Women vs. Men	Incidence of MI (%)	P value	Incidence of Stroke (%)	P value
**Entire cohort**	Women	5.2%	0.221	7.9%	0.212
	Men	5.9%		7.0%	
**Subgroup**					
**MT alone**	Women	5.4%	0.938	6.9%	0.535
	Men	5.4%		6.2%	
**PCI**	Women	6.7%	0.063	7.4%	0.110
	Men	8.7%		5.9%	
**CABG**	Women	2.6%	0.873	9.5%	0.865
	Men	2.5%		9.3%	

CABG, coronary artery bypass grafting; PCI, percutaneous coronary intervention; MT, medical therapy; MI myocardial infarction.

Table S5. Univariable and Multivariable Cox Proportional Hazards Model for All-Cause Death.

**Parameters**	**Univariable Cox model**		**Multivariable Cox model**	
	**HR (95% CI)**	***P* value**	**HR (95% CI)**	***P* value**
Female Sex	1.12 (0.98–1.27)	0.088	0.78 (0.67–0.90)	0.001
Age	1.07 (1.06–1.08)	<0.001	1.04 (1.04–1.05)	<0.001
BMI	0.94 (0.92–0.96)	<0.001	1.01 (0.99–1.03)	0.342
Previous MI	1.50 (1.35–1.67)	<0.001	1.26 (1.11–1.43)	0.001
Previous stroke	1.92 (1.66–2.22)	<0.001	1.42 (1.20–1.67)	<0.001
Hypertension	1.10 (0.98–1.23)	0.106	–	–
Hyperlipidaemia	0.81 (0.73–0.91)	<0.001	0.87 (0.77–0.98)	0.020
Diabetes	1.47 (1.32–1.63)	<0.001	1.49 (1.32–1.67)	<0.001

COPD	1.97 (1.34–2.90)	0.001	0.89 (0.57–1.40)	0.622
PAD	1.59 (1.33–1.90)	<0.001	1.14 (0.93–1.38)	0.206
Previous PCI	0.98 (0.84–1.15)	0.810	0.92 (0.77–1.10)	<0.001
Previous CABG	1.30 (1.01–1.67)	0.040	0.99 (0.73–1.34)	0.346
Current/former smoker	1.06 (0.96–1.19)	0.250	–	–
Clinical presentation (ACS vs. SAP)	1.39(1.24–1.55)	<0.001	1.25 (1.10–1.42)	<0.001
LVEF	0.95 (0.95–0.96)	<0.001	0.97 (0.97–0.98)	<0.001
TC	0.99 (0.94–1.04)	0.600	–	–
HDL-C	0.91 (0.74–1.12)	0.384	–	–
LDL-C	1.02 (0.96–1.08)	0.479	–	–
CCr	0.98 (0.97–0.98)	<0.001	0.99 (0.98–0.99)	<0.001
Left main involvement	1.10 (0.97–1.24)	0.149	1.01 (0.88–1.17)	0.869

SYNTAX score	1.01 (1.01–1.02)	<0.001	1.01 (1.01–1.02)	<0.001
Treatment strategies				
PCI vs. MT	0.36 (0.32–0.41)	<0.001	0.51 (0.44–0.59)	<0.001
CABG vs. MT	0.36 (0.32–0.42)	<0.001	0.40 (0.34–0.47)	<0.001

CI, confidence interval; HR, hazard ratio; meanings of other abbreviations are identical to those in Table S2.

Table S6. The Effect of Sex on Long-term MI and Stroke in Patients with TVD in the Entire Cohort and Treatment Strategy Stratification.

Clinical Outcomes	Women vs. Men	Adjusted HR (95% CI)	P value
**MI**	Entire Cohort	0.942 (0.730–1.215)	0.645
	MT alone group	0.994 (0.615–1.606)	0.980
	PCI group	0.837 (0.595–1.177)	0.306
	CABG group	1.262 (0.662–2.407)	0.480
	**P for interaction**		0.532
**Stroke**	Entire Cohort	1.101 (0.896–1.354)	0.359
	MT alone group	1.236 (0.821–1.861)	0.311
	PCI group	1.025 (0.722–1.457)	0.888
	CABG group	1.117 (0.800–1.558)	0.517
	**P for interaction**		0.738

CABG, coronary artery bypass grafting; PCI, percutaneous coronary intervention; MT, medical therapy; CI, confidence interval; HR, hazard ratio; MI myocardial infarction.
